# Calculous Diseases in China

**Published:** 1853-04

**Authors:** 


*Calculous Diseases in China.*—The frequency of the admission of calculous disorders into this hospital (Canton) is another proof of the erroneousness of the belief which once prevailed in regard to the existence of these diseases in the Eastern tropical climates. This opinion was based principally upon the testimony of Mr. Scott, an English writer, who remarked that "the formation of stone in the urinary bladder is nearly unknown between the tropics. I have, indeed, not met with a single instance of it, although I have known some cases where such a disease was imported and not removed by climate." Mr. Smith, in his *Statistical Inquiry into the Frequency of Stone in the Bladder* (Med. Chir. Trans. vol. xi.) quotes the authority of Mr. Hutchinson, that the operation for stone was never performed in the British foreign possessions. The cause of this freedom from calculous diseases in such regions was attributed to the greater activity of the cutaneous system and proportionable decrease of the urinary secretion. English surgeons, however, attached to the East India service, have not only proved the utter groundlessness of this opinion, but, by the publication of their cases, show that it is a very common affection, its frequency varying, as in other countries, with localities. Mr. Burnard (*Trans. of Med. and Phys. Soc. of Calcutta*, vol. v.) gives a table of thirteen operations, of which twelve were performed in 1830, at the Hospital of Benares. Mr. Brett reported in the same volume seven operations, of which number three died of tetanus. In the subsequent volumes of the Transactions a large num- of cases is recorded. In the 4th volume, Mr. Lindsay reports an operation by a native, and remarks that this surgeon said he had operated 150 times. In the *Trans. Med. and Phys. Soc. of Bombay*, Dr. Arnott speaks of calculous diseases as common in the villages of his vicinity. —*N. Y. Journal of Medicine.*

